# Bonobo mothers have elevated urinary cortisol levels during early but not mid or late lactation

**DOI:** 10.1007/s10329-022-01044-7

**Published:** 2022-12-24

**Authors:** Niina O. Nurmi, Ruth Sonnweber, Oliver Schülke, Liza R. Moscovice, Tobias Deschner, Gottfried Hohmann

**Affiliations:** 1 Department of Behavioral Ecology, JFB Institute for Zoology/Anthropology, University of Goettingen, Göttingen, Germany; 2 Interim Group Primatology, Max Planck Institute for Evolutionary Anthropology, Leipzig, Germany; 3 Department of Behavioural and Cognitive Biology, University of Vienna, Vienna, Austria; 4 Research Group Social Evolution in Primates, German Primate Center, Leibniz Institute for Primate Research, Göttingen, Germany; 5 Institute of Behavioral Physiology, Leibniz Institute for Farm Animal Biology, Dummerstorf, Germany; 6 Max Planck Institute for Evolutionary Anthropology, Leipzig, Germany

**Keywords:** Reproductive stage, *Pan paniscus*, Energetic stress, Circadian rhythm, Glucocorticoids

## Abstract

In mammals, the costs of reproduction are biased towards females. Lactation is particularly energetically expensive, and behavioral and physiological data indicate that maternal effort during lactation induces energetic stress. Another source of stress in females is male aggression directed towards them when they are cycling. Evaluating the costs of reproduction in wild and mobile animals can be a challenging task, and requires detailed information on state-dependent parameters such as hormone levels. Glucocorticoid (GC) levels are indicative of nutritional and social stress, and are widely used to assess the costs of reproduction. We investigated variation in urinary levels of cortisol, the main GC in female bonobos (*Pan paniscus*), between and within reproductive stages. Female chimpanzees (*Pan troglodytes*), the closest living relative of the bonobos, are often exposed to intense aggression from males, which causes a significant rise in their cortisol levels during the phase of their maximum fecundity. In bonobos, males compete for access to fertile females, but aggressive male mating strategies are absent in this species. Therefore, we expected that GC levels of cycling female bonobos would be lower than those of lactating females. Due to the long period of offspring care in bonobos, we expected that GC levels would remain elevated into the late stage of lactation, when immatures gain body weight but may still be nursed and carried by their mothers. We found elevated urinary GC levels only during the early stage of lactation. The GC levels of cycling females did not differ from those in the mid or late lactation stage. Behavioral strategies of female bonobos may allow them to compensate for the elevated energetic demands of lactation and prolonged maternal care.

**Supplementary Information:**

The online version contains supplementary material available at 10.1007/s10329-022-01044-7.

## Introduction

Among mammals, females carry the metabolic costs of gestation and lactation, and therefore reproduction is energetically exceptionally expensive for them [Gittleman and Thompson 1988; humans (*Homo sapiens*) (Butte and King 2005; Ponzer et al. 2014)]. In primates, the combined length of the gestation and lactation periods tends to exceed that of other mammalian species of similar body mass (Dufour and Sauther 2002) and, unlike other mammals, primate mothers carry their offspring for extended periods of time, which increases their energetic costs during the period of lactation (Ross 2001). One quantitative approach to exploring the influence of reproductive state on female energy status is to measure variation in physiological metabolic markers, such as glucocorticoids (GCs) or thyroid hormones, across different female reproductive phases (Emery Thompson 2013, and references therein). Increased production of GCs is an adaptive physiological response to higher energy requirements (Karatsoreos and McEwen 2010; Romero and Beattie 2021). Data from various mammalian species have revealed that lactating females have higher GC levels than non-lactating females [e.g., spotted hyenas *Crocuta crocuta* (Goymann et al. 2001); red deer (*Cervus elaphus*) (Pavitt et al. 2016); red squirrels (*Sciurus vulgaris*) (Dantzer et al. 2016)]. A similar pattern in CG levels has been found in studies on primate species that compared cycling with lactating females [chacma baboons (*Papio ursinus*) (Weingrill et al. 2004); chimpanzees (*Pan troglodytes*) (Emery Thompson et al. 2012; but see Emery Thompson 2010)]. However, energetic demands seem to vary not only between but also within reproductive states; in primates, GC levels reach maxima during early lactation [e.g., forest guenons (*Cercopithecus mitis*) (Foerster et al. 2012); rhesus macaques (*Macaca mulatta*) (Dettmer et al. 2015); Assamese macaques (*Macaca assamensis*) (Touitou et al. 2021a); humans (Brunton et al. 2008)]. During early lactation, infant milk intake is particularly high, and the infant has to be carried most of the time by its mother [e.g., chimpanzees (Emery Thompson et al. 2012)].

Among primates, bonobos (*Pan paniscus*) are of particular interest for the study of the relationship between reproductive status and GC levels. One factor likely to affect the physiological status of females is exposure to intraspecific aggression (Creel et al. 2013). In non-human primate societies that are both polyandrous and polygynous, cycling females with a tumescent perineal swelling, especially around the peri-ovulatory period, are often exposed to higher rates of male aggression as compared to other phases of their reproductive cycle or non-sexually receptive phases due to pregnancy or lactation [Smuts and Smuts 1993; hamadryas baboons (*Papio hamadryas*) (Swedell et al. 2014; Malamuth et al. 2005); chacma baboons (Baniel et al. 2017)]. Increased exposure to male aggression can induce an increase in GC excretion [e.g., East African chimpanzees (Wrangham 2002; Muller et al. 2007; Emery Thompson et al. 2010)]. At Kanyawara, Kibale, cycling female chimpanzees were found to have higher GC levels during their peak swelling phase than females that were cycling but were not in full tumescence, were non-swollen, were in early lactation, or were in the late phase of lactational amenorrhea [Emery Thompson et al. (2020); but see Emery Thompson et al. (2010) for a report of higher urinary cortisol levels in estrous and lactating females compared to non-estrous females]. Also in bonobos, male-male aggression rates increase in the presence of sexually attractive females (Hohmann and Fruth 2003; Surbeck et al. 2012; Ryu et al., in press), but unlike male chimpanzees, bonobo males do not direct aggression towards females (Hohmann and Fruth 2003). Because of the absence of coercive male mating strategies in bonobos, we did not expect to find elevated GC levels in cycling bonobo females in comparison to females in other reproductive phases. 

The long period of somatic growth and slow social maturation in bonobos that results in immatures relying on maternal support for a longer period of time than in other mammal species, also renders them an interesting species to study with respect to the association between reproduction and GC levels. Newborn bonobos suckle around twice an hour, and 1- to 6-month-old bonobos may even nurse three times an hour; the nursing rate decreases to twice hourly between 6 months and 1 year of age (Weaver 1997). Weaning age in wild bonobos is around 4 to 5 years (Kuroda 1989), although reports exist of even older offspring that may not have been fully weaned (de Lathouwers and Van Elsacker 2006; Johnson 1997). During the later period of lactation, bonobo females spend more time traveling and feeding than in the early stage (Lee et al. 2021), and thus may face increased energetic demands at that time. Moreover, the transportation of infants is a particularly energetically expensive form of maternal effort, and is likely to impose a metabolic burden on lactating females [yellow baboons (*Papio cynocephalus*) (Altmann and Samuels 1992)]. Only at the age of 7 years are bonobos independent of their mothers in regard to feeding and movement (Lee et al. 2020). Indirect evidence of the effects of the long period of maternal dependency in bonobos is provided by a recent study that shows that the birth of a sibling is associated with a severe and lasting stress response in older siblings, even in adolescent ones (Behringer et al. 2022), which may indicate that the shift of maternal care towards the younger sibling negatively impacts the older offspring. To summarize, bonobos may represent a special case amongst mammals due to the lack of male coercion and aggression towards fertile females, and the particularly long period of intense offspring care, which may incur an energetic cost in lactating females. As a consequence of this, elevated GC levels are expected only in lactating females and not in cycling females.

To test these predictions, we measured urinary levels of cortisol, the main GC in our study species, in cycling and lactating female bonobos at the LuiKotale field site, Democratic Republic of the Congo. We did not include pregnant females in the study, as it is difficult to distinguish between maternally and fetally induced GCs [e.g., humans (Mastorakos and Ilias 2009)]. We split the lactation period into stages based on milestones of infant physical and behavioral development that are likely to coincide with differing metabolic loads for nursing mothers in wild bonobos (Lee et al. 2020, 2021). (1) From birth to 6 months postpartum (early lactation), when an infant’s motoric skills are poorly developed and it spends most of its time in physical contact with its mother. (2) From 6 months after the birth of the infant until it reaches 2 years of age (mid lactation), when the time the infant spends away from the mother increases and is indicative of independent travel. (3) From when the infant is 2 years old until it reaches 4 years of age (late lactation), during which period the infant rides less on its mother’s back and progressively wanders further away from its mother, and engages in social play and sexual activities (Hashimoto 1997). Preliminary data suggest that the stage of nutritional weaning is reached by the age of 4 years (Oelze et al. 2020; Kuroda 1989; but also see Lee et al. 2020). 

A general prediction is that urinary cortisol levels of cycling female bonobos are lower than those of females at the early lactation stage. This prediction is based on the observation that male bonobos do not employ coercive mating strategies that would constitute a stressor for cycling fecund females, and takes into consideration the general mammalian pattern that the phase of early lactation is a particularly demanding one energetically. For the other phases of lactation, we predict that the metabolic demands and related urinary cortisol levels will remain elevated throughout all phases of lactation (early, mid, and late). Underlying our prediction is the observation that bonobo mothers not only nurse their offspring, but also intensively care for them (such as carrying the infant) for at least the first 4 years of an infant’s life, which is likely energetically very demanding. Finally, we predict that urinary cortisol levels in female bonobos follow typical daily fluctuations independent of reproductive stage, with higher levels in the morning and decreasing levels as the day progresses.

## Methods

### Ethics statement

Our protocols and methods followed a strict non-contact, non-invasive procedure. The Institut Congolaise pour la Conservation de la Nature (ICCN) granted us permission to conduct research at LuiKotale, Salonga National Park, Democratic Republic of Congo (0683/ICCN/DG/ADG/014/KV/2012). Permits for exporting the urine samples from the Democratic Republic of Congo were issued by the ICCN (0521/ICCN/DG/CWB/05/01/2014). The German ministry for social affairs and consumer protection issued permits for importing the samples.

### Study site and subjects

The LuiKotale field site is located close to the south-western edge of Salonga National Park, in the Democratic Republic of Congo. The study site (2º47’S, 20º21 E) is situated in a continuous bloc of equatorial rainforest mainly comprising lowland tropical forest. Rainfall tends to be seasonal, with a long dry season from June to August, and a short dry season around February (Bessone et al. 2021). Our research was conducted between February 2012 and August 2014. During this period, we collected data on the fully habituated Bompusa West community, which consisted of five adult and two subadult males, and 16 adult and three subadult females. Of the 16 adult females, 13 were parous females and three were nulliparous. We define a nulliparous female as a non-adolescent female that has reached reproductive maturity, but has not given birth. Female bonobos are estimated to reach reproductive maturity at around 13 years of age (de Waal 1997). The 14 adult female subjects included in this study were considered to have been residents of the community from the onset of the study. Urine of two of the nulliparous females could not be sampled sufficiently and thus was excluded from the analyses. As the dates of birth of the adult females involved in our study were unknown, chronological age could not be reliably estimated in the models (see below).

### Urine sample collection

We collected 544 urine samples opportunistically from the 14 female bonobos (for details on female age, parity, sex of offspring, etc., see Supplementary material S1) throughout the study period (mean ± SD = 37 ± 15 samples per individual, range = 13–66). Most of the samples were collected from the underside of a large leaf. For the other samples, the urine was pipetted directly from foliage on the ground (*n* = 35) or from a frisbee covered with a plastic bag (*n* = 62). Samples were only collected on condition that they had not come into contact with urine from another individual, or with feces. The samples were stored in liquid nitrogen containers on the day of collection in the field and later transported on dry ice to the Max Planck Institute for Evolutionary Anthropology, Leipzig, Germany, where they were stored at − 20 °C on arrival until hormone analysis.

### Cortisol extraction and measurement

We extracted cortisol from urine samples according to the method described by Hauser et al. (2008) with the exception that testosterone-d_3_ (T2655, Sigma-Aldrich) not prednisolone was used as an internal standard for the determination of extraction efficiency, as the latter eluted at the same retention time as an unknown substance. Before extraction, the samples were thawed at room temperature, mixed for 10 s in a vortexer (VX-2500, VWR), and centrifuged for 10 min at 4400 r.p.m. (Multifuge, Heraeus). After addition of an internal standard mixture to each sample, the steroid glucuronides were deconjugated using enzymatic hydrolysis with ß-glucuronidase from *Escherichia coli* (G7646, Sigma), followed by a solvolysis step using ethyl acetate/sulfuric acid to cleave steroid sulfate conjugates. We measured urinary cortisol levels using liquid chromatography-tandem mass spectrometry (Waters Acquity UPLC coupled to a Xevo TQ-S MS with an Z-spray ESI interface) in a method adapted from Hauser et al. (2008). Control samples with a known concentration of cortisol were included in each measurement batch. Control sample measurements were considered acceptable when they deviated less than 15% from the known cortisol concentration. Extraction efficiency was calculated based on the recovery of the internal standard, testosterone-d_3_. Our exclusion threshold for internal standard loss was measurement deviation of less than 70% from the concentration of the internal standard. We excluded samples when loss of the external standard was too high (*n* = 19). We corrected for variation in urine concentration by measuring the level of creatinine in each sample by Jaffe’s colorimetric method (Bahr et al. 2000) and indexing cortisol levels to creatinine. Thus, all cortisol measurements are reported as nanograms cortisol per milligram creatinine. Urine samples with less than 0.05 mg creatinine/ml were excluded from the analysis (*n* = 4). We used this threshold because a very low concentration of creatinine indicates that there is a high chance that the sample is diluted with rain water, and thus the cortisol level is no longer meaningful.

### Reproductive state

For each day, we assigned each female to one of the following reproductive states: early lactation, from parturition until her infant reached 6 months of age (*n* = 68 samples); mid lactation, from 6 months after parturition until her infant reached 2 years of age (*n* = 145 samples); late lactation, from when her infant reached 2 years of age until it reached 4 years of age (*n* = 128 samples); and cycling, when a female had an infant(s) older than 4 years of age (possibly including a post-reproductive female and nulliparous females) (*n* = 82 samples). This classification is based on preliminary assessments of weaning age of immatures from the same bonobo population (Oelze et al. 2020). Because of their low incidence (*n* = 30 samples) and possible large impact with respect to the measured cortisol levels [e.g., humans (Duthie and Reynolds 2013)], we excluded samples from pregnant females, for whom pregnancy was assessed retrospectively from parturition and confirmed by pregnancy test for eight of the 14 individuals. In a second analysis, we used data from lactating females only (excluding cycling females). The energetic status of lactating females may vary discretely over time due to changes in environmental or social factors, which may not be accounted for by a categorical variable. Therefore, instead of comparing differences in GC across the three lactational states defined above, we used chronologic age of offspring (number of days from birth) as a predictor variable in the models. Transforming lactation into a discrete variable allowed us to track urinary cortisol levels during the course of the lactation period.

### Statistical analyses

Following the exclusion of 53 urine samples due to pregnancy of the females from which they had been collected (*n* = 30), external standard loss (*n* = 19), or low creatinine level (*n* = 4), we were able to assess the influence of reproductive phase on cortisol levels across 423 urine samples, by fitting a linear mixed model (LMM) with a Gaussian error structure (Baayen 2008). We included time of sample collection as a predictor because cortisol levels usually decrease from the morning throughout the day [bonobos (Verspeek et al. 2021)], although this circadian pattern has not yet been explicitly demonstrated for wild female bonobos. The response variable, cortisol level (standardized for creatinine), was log transformed to obtain a more symmetrical distribution. Reproductive state was entered as a categorical test variable, with four levels: cycling, early lactation, mid lactation, late lactation.

We included female identity as a random effect to control for repeated measures (Schielzeth and Forstmeier 2009; Barr et al. 2013). We were not able to add a random slope of reproductive state or time of day within female identity because this led to stability issues with the model, caused by singularity (Darlington and Hayes 2017, p. 534). In other words, the random effect structure had to be simplified due to the lack of variation within females to account for each level of reproductive state or time of day. The fact that our initial models did not converge was most likely due to the limited number of urine samples per female for the different reproductive states. The variance inflation factor (VIF) was determined for the standard linear model, excluding interaction and random effects, to check for potential multicollinearity issues among the predictors by using the function vif [R package car (Fox and Weisberg 2011)]. Collinearity was not detected (maximum VIF = 3). Significance of the full model (Forstmeier and Schielzeth 2011) was tested using a likelihood ratio test (Dobson 2002) by comparing it with the respective null model containing only the random effect. We tested the significance of each test variable (reproductive state and time of sample day) one at a time (Barr et al. 2013) using the likelihood ratio test in R.

A second LMM was run using 347 samples from lactating females as a post hoc test to check for the relationship between urinary cortisol and lactation duration, with log-transformed cortisol level as the response variable. We entered lactation days, defined as the number of days from birth of the infant until the day of sample collection, as a continuous variable. The sampling time was also included as a control variable to account for circadian variation in cortisol excretion. As random effects, a random slope was added for lactation state and a random intercept for female identity (Barr et al. 2013). There were no issues with collinearity (maximum VIF = 1.00). We tested the significance of the model by comparing the full model (Forstmeier and Schielzeth 2011) with the respective null model (random effect only) using a likelihood ratio test (Dobson 2002). On the condition that the full model was significantly different from the null model, the significance of the main effect was tested by comparing the full model with a reduced model in which the main effect of lactation days was removed.

All LMMs were run in R Studio version 1.3.959 (RStudio Team 2020) using the function lmer [R package lme4 (Bates et al. 2015)] with the threshold for statistical significance set at *P* = 0.05. We visually inspected quantile–quantile plots and distribution of residuals plotted against fitted values to check that the assumptions of normally distributed and homoscedastic residuals were met; there was no indication of deviation from these assumptions.

## Results

**Fig. 1:**
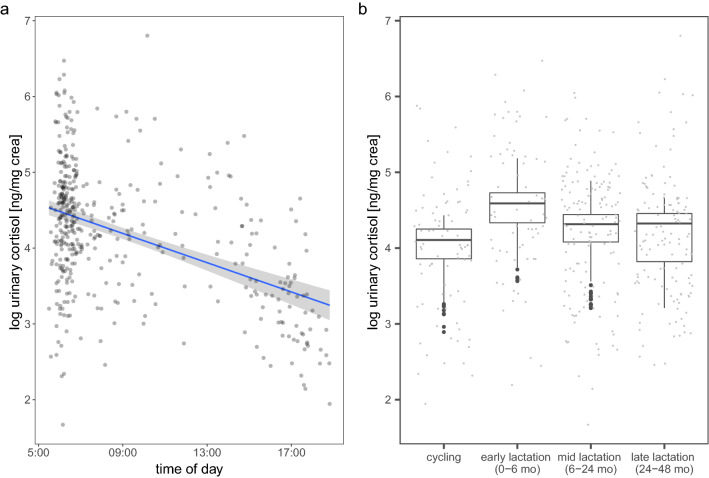
The effect of time of day of sample collection on urinary cortisol levels [ng/mg creatinine (*crea*)], shown on a log scale (**a**). Urinary cortisol levels are highest in the morning then decrease throughout the day. Boxplot showing log-transformed urinary cortisol levels (ng/mg crea) of female bonobos in different reproductive stages (**b**). Females had highest urinary cortisol levels during early lactation (from an infant’s birth up until 6 months of age). There was no statistically significant difference between urinary cortisol levels of females that were cycling, in mid lactation or in late lactation

Average cortisol levels of individual female bonobos ranged from 80.60 to 192.77 ng/mg creatinine. As expected based on the diurnal cortisol rhythm in other primates, cortisol was highest in the morning and decreased throughout the day (estimate ± SE = − 0.74 ± 0.07, *P* < 0.001; Table 1; Fig. 1a). To test for the costs of reproduction on individual female bonobos, we examined whether variation in cortisol levels (range 5.31–899.35 ng/mg creatinine) was explained by reproductive state (cycling, early lactation, mid lactation, late lactation). The full model, which contained all predictor variables and random effects, differed significantly from the null model containing only the random effects (χ^2^ = 107.84, *df* = 4, *P* < 0.001; detailed model outcomes are shown in Supplementary material S2). Therefore, we tested for effects of individual predictor variables (see Table 1). Female reproductive state had an impact on urinary cortisol levels (χ^2^ = 16.58, *df* = 3, *P* < 0.001), with the highest levels occurring during early lactation (reference level of pairwise comparison; Table 1; Fig. 1b). Pairwise post hoc comparisons showed that urinary cortisol levels in females were significantly higher when they were in early lactation than when they were cycling (estimate ± SE = 0.59 ± 0.16, *P* = 0.001), or were in mid lactation (estimate ± SE = 0.32 ± 0.11, *P* = 0.003) or in late lactational periods (estimate ± SE = 0.41 ± 0.15, *P* = 0.003). None of the other reproductive state categories differed in regard to urinary cortisol levels.

**Table 1:** Model results for the linear mixed model testing for the effects of time of day and reproductive state (cycling, early lactation, mid lactation, and late lactation) on urinary cortisol levels in female bonobos

	Estimate	SE	*P*-value
Intercept	5.282	0.132	
Time of day (*z*-transformed)	− 0.742	0.073	< 0.001
Reproductive state (cycling)	− 0.592	0.148	< 0.001
Reproductive state (mid lactation)	− 0.317	0.113	
Reproductive state (late lactation	− 0.409	0.137	

Reproductive states (cycling, mid lactation, late lactation) were tested with early lactation as the reference category (positive estimates indicate higher values in relation to early lactation, negative estimates indicate lower values in relation to early lactation). Urinary cortisol levels were highest during early lactation

**Fig. 2:**
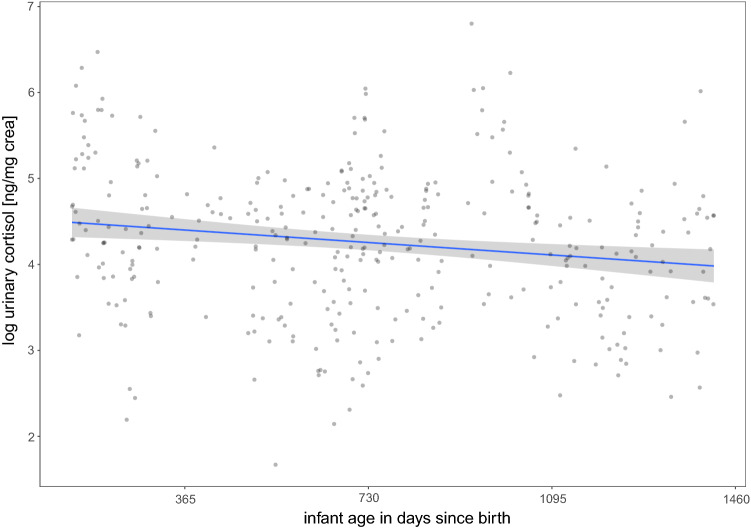
Urinary cortisol levels (ng/mg crea) of lactating female bonobos as predicted by the length of their lactational period (age of offspring in days) at the time of sampling. Urinary cortisol levels were highest at the onset of lactation and decreased slowly but steadily as the offspring matured

We then conducted a second analysis using samples from lactating females only, to account for discrete changes in female energetic condition throughout the lactational period, by fitting a model to test for the relationship between lactating female urinary cortisol level and offspring age (in days). The full and null model differed significantly (χ^2^ = 72.25, *df* = 2, *P* < 0.01). Contrary to our predictions, females at earlier stages of lactation had higher urinary cortisol levels than females at later stages (estimate ± SE = − 0.40 ± 0.13, *P* < 0.01; Fig. 2).

## Discussion

The goal of this study was to explore the costs of reproduction of wild female bonobos within and between different reproductive stages, using measures of urinary cortisol as a proxy for energetic and social stress. GCs are the primary hormones of the hypothalamo-pituitary-adrenal (HPA) axis, and activation of the HPA axis can be viewed as a one-size-fits-all reaction to many external or internal challenges to homeostasis (Beehner and Bergman 2017). With the exception of when GCs are produced in response to stress, their excretion follows a diurnal pattern [e.g., rhesus macaques (Plant 1981; Smith and Norman 1987)], and our results are consistent with these and other studies based on urinary cortisol levels [e.g., bonobos (Verspeek et al. 2021); chimpanzees (Muller and Lipson 2003); long-tailed macaques (*Macaca fascicularis*) (van Schaik et al. 1991); humans and western lowland gorillas (*Gorilla gorilla gorilla*) Czekala et al. 1994)], as we found a steady decrease in urinary cortisol levels from the morning onwards, throughout the day.

The measurement of hormones levels is increasingly used to explore the energetic costs of reproduction (Beehner and Bergman 2017). While the energetic costs of milk production in humans are thought to be buffered by the mother’s energy status (Ellison 2003), data from nonhuman primates [Dufour and Sauther 2002; Hinde and Milligan 2011; chacma baboons (Weingrill et al. 2011); chimpanzees (Emery Thompson et al. 2012); forest guenons (Foerster et al. 2012); Assamese macaques (Fürtbauer et al. 2014)] and other mammals [e.g., North Atlantic right whale (*Eubalaena glacialis*) (Hunt et al. 2006); red squirrel (Fletcher et al. 2013)] indicate that lactation is metabolically particularly challenging as compared to non-lactational phases, and that mammalian species have developed, to a lesser or greater extent, coping strategies to buffer the costs of lactation and offspring care. For instance, seasonal breeding may be one way of buffering the energetic demands of lactation, by restricting the period of intensive offspring care to a time when resources are more available and easy to access [e.g., topi (*Damaliscus lunatus jimela*) and warthog (*Phacochoerus africanus*) (Ogutu et al. 2010)]. Some species are able to compensate for the costs of lactation by reliance on energy reserves and/or modification of activity profiles [e.g., *Papio* spp. (Gesquiere et al. 2018)]. 

However, some studies counter the prevailing view of lactation as a particularly energetically costly phase; for example, the energetic demands of lactation, after the first phase, may be similar to those due to food deprivation or seasonal environmental factors [yellow baboons (Gesquiere et al. 2008); olive baboons (Lodge et al. 2013)]. Moreover, when energetic constraints are buffered by food provisioning (e.g., in captive colonies), lactation can be of low cost [olive baboons (Garcia et al. 2009)]. In the case of bonobos, the variation in urinary cortisol levels across different reproductive stages found here suggests that the first 6 months after parturition are energetically more demanding than later periods of lactation. This result was unexpected for two reasons. First, the demand for milk is expected to increase with offspring age. Accordingly, the energetic investment in milk production should increase with offspring age until immatures start feeding on other foods. In captive bonobos, feeding on other foods starts when infants are between 3 and 8 months of age (Fagan 1997; Hübsch 1970; Jordan 1977; Kano 1992; Weaver 1997), but in wild bonobos this is expected to occur when infants are older. Preliminary data suggest that immatures are nutritionally weaned at the age of 4 years (e.g., De Lathouwers and Van Elsacker 2005; Oelze et al. 2020) but more detailed data suggest that, in a wild population, this state of independence is reached later [Oelze et al., personal communication; for accounts of this, also see Johnson (1997)]. Second, as in other primates, female bonobos carry their infants during the first years of life, and although carrying time decreases as the infants mature (Lee et al. 2020), carrying immatures during mid (infant > 0.5 years of age) or late lactation (infant > 2 years of age) is expected to be energetically more demanding compared to in early lactation simply because the infants are heavier. In addition, there is evidence that female bonobos occasionally simultaneously care for two offspring, which is likely to substantially increase energetic costs (Furuichi et al. 2014). 

Another study of LuiKotale bonobos (Lee et al. 2021) revealed differences in activity patterns of lactating females in different stages of lactation, where mothers of older infants spent more time traveling and feeding as compared to mothers with younger infants (Lee et al. 2021). Thus, mothers in the early lactation phase may be more likely to travel alone or in smaller parties (Moscovice et al. 2017; Surbeck et al. 2021), and as a consequence, their energy expenditure may be reduced. In addition to reducing the energetic costs associated with travel, females with small infants may prefer to travel separately to avoid feeding competition with other females (Nurmi et al. 2018). Feeding in larger parties can reduce rates of food intake, shorten the time until a given food patch is depleted, and increase travel time [e.g., yellow baboons (Markham and Gesquiere 2017); long-tailed macaques (van Schaik et al. 1983)], and may therefore not be sustainable for mothers with small infants [e.g., chimpanzees (Wrangham 2000)]. Another possible reason for females to avoid large parties is the risk of male aggression towards their infants. At LuiKotale, older immatures are sometimes the target of aggression from adult males, and the risk of this occurring is particularly high when their mothers have given birth to another (younger) sibling (Hohmann et al. 2019). Adult males are more likely to travel in larger parties, and mothers with newborns and older immature offspring may avoid mixed parties to protect the latter from male aggression. Although there are benefits associated with the avoidance of social activities, for example reduced resource competition and avoidance of the energetic costs associated with lengthy periods of travel (particularly when a mother is carrying an infant that is unable to hold on by itself), reduced gregariousness is likely to deprive lactating females of access to meat [for meat monopolization by females, see Fruth and Hohmann (2018)] or high-quality plant foods that are usually consumed communally in large parties, as well as access to potential female partners for various forms of cooperation, including grooming and food sharing (Tokuyama and Furuichi 2016). Thus, a potential lack of high-quality food sources due to social avoidance may hinder effective energetic compensation (through caloric intake) of energy expenditure by females related to the early phase of lactation.

Interpretation of the results of this study is hampered by the lack of information on reproductive physiology (e.g., when did ovulation occur and when was a female not in her fertile period) and resource availability (e.g., which foods were available and to what extent, and what was the caloric value of the different plant types consumed). As regards reproductive physiology, the time when mothers resume cycling can be used to more accurately determine when weaning occurs. However, in the case of bonobos this is complicated, as females may show sexual swellings a few months after parturition (Heistermann et al. 1996), but swelling cycles do not coincide with ovulation in this species, and the detection of changes in fecundity requires a high sampling rate to measure hormonal changes (Douglas et al. 2016), which is challenging under field conditions. Second, the climate at LuiKotale is seasonal (Bessone et al. 2021), and it is reasonable to assume that this affects the abundance and quality of plant foods a different times of the year. Thus, these types of data should be collected and analyzed in future longitudinal studies. Furthermore, data on food consumption could be collected so that energetic buffering of the costs of reproduction can be studied. At LuiKotale, Bonobos hunt other mammals for food, and as adult females monopolize the sharing out of meat (Fruth and Hohmann 2018), meat may constitute a major source of their high caloric food intake. In our data set, the rate of meat-eating events was too low to be included as a predictor, but future studies using longitudinal datasets may be able to shed more light on the role of meat eating in lactating females. Finally, one caveat of our study is limitations in our statistical analyses due to sample size. While the sample density and number of females were acceptable for data collected in the wild, it was not possible to account for random slopes of time of data collection and reproductive phase within individuals. Also, parameters that potentially affect lactation and suckling trajectories over time and differ between reproductive events (female age, parity, the sex of the offspring, body mass, energy balance, season, to name but a few) could not be accounted for in our statistical analyses. This lack indicates once again the importance of large, longitudinal datasets. Thanks to the efforts of many, such datasets may become available in the foreseeable future for various non-human primates at field sites, which will allow researchers to gain more insight into physiological mechanisms and behavioral coping strategies associated with female reproductive costs.

Mammals can adopt different strategies to compensate for the metabolic costs of lactation, such as a reduction in physical activity, an increase in caloric intake, and increased reliance on stored body fat [humans and non-human primates (Dufour and Sauther 2002); grey seals (*Halichoerus grypus*) (Shuert et al. 2020)], and/or changes in gut morphology and contents [e.g., rodents (*Octodon degus*) (Naya et al. 2008)]. Female chimpanzees, for instance, feed on higher quality foods during pregnancy and lactation (Murray et al. 2009), and Assamese macaques feed for longer periods, consuming a fiber-rich diet, at the expense of periods of resting at gestation and late lactation (Touitou et al. 2021b). Female moose (*Alces alces*) move to birth sites that contain twice the amount of available forage, such as willow (*Salix*), compared to random ranging sites (Bowyer et al. 1999). During advanced periods of lactation, female bonobos feed for longer and spend more time traveling than at other reproductive periods (Lee et al. 2021), and both strategies are likely to compensate for increased energy demands through the exploitation of food resources that are of higher caloric value. Apart from the first 6 months of lactation, when levels of urinary cortisol excretion increase, female bonobos appear to have strategies to cope with the costs of lactation that do not trigger prolonged periods of heightened cortisol levels. This puts bonobos in line with humans and most other non-human primates, as well as many other (large-bodied) placental mammals, with respect to energetic patterns associated with reproduction.

## Supplementary Information

Below is the link to the electronic supplementary material.

Supplementary file1 (DOCX 21 KB)

Supplementary file2 (CSV 53 KB)

## Data Availability

Data are available from the supplementary materials.
