# Supplementary material for: Bonobo mothers have elevated urinary cortisol levels during early but not mid or late lactation
Source: Primates. 2022 Dec 24;64(2):215–25. doi: 10.1007/s10329-022-01044-7 (PMC10006042; doi:10.1007/s10329-022-01044-7)
Supplement: Supplementary file 1 — Supplementary file1 (DOCX 21 KB) [file 10329_2022_1044_MOESM1_ESM.docx]

Supplementary material: **Bonobo mothers have elevated urinary cortisol levels during early but not mid or late lactation**

**S1: Information on individual female bonobos:**

| **Female ID** | **Age** | **Parity** | **Sex of offspring** | **Season of sampling** |
| --- | --- | --- | --- | --- |
| Djulie | young | nulliparous | - | 2012/2013/2014 |
| Gwen | mid | multiparous | male | 2012/2013/2014 |
| Iris | old | multiparous | male | 2012/2013/2014 |
| Luna | young | primiparous | female | 2013/2014 |
| Martha | old | multiparous | - | 2012/2013/2014 |
| Nina | mid | primiparous | female | 2012/2013/2014 |
| Olga | old | multiparous | male | 2012/2013/2014 |
| Paula | old | multiparous | female | 2012/2013/2014 |
| Polly | young | nulliparous/primiparous | female | 2012/2013/2014 |
| Rio | old | multiparous | female | 2012/2013/2014 |
| Susi | mid | nulliparous/primiparous | female | 2012/2013/2014 |
| Uma | mid | multiparous | male | 2012/2013/2014 |
| Wilma | mid | primiparous | female | 2012/2013/2014 |
| Zoe | old | multiparous | male | 2012/2013/2014 |

**S2: Details of model results:**

Comparison of the three competing “categorical” models: weighted AICc scores indicate that the full model fits the data best.

| **MODEL 1** | **AICc** | **delta AICc** | **expAICc** | **wAICc** |
| --- | --- | --- | --- | --- |
| full | 966.7828 | 0 | 1 | 0.99 |
| excluding reproductive state | 977.1888 | 10.406 | 0.00550004 | 0.01 |
| excluding time of sample collection | 1057.46 | 90.6772 | 2.0403E-20 | 0.00 |
|  |  |  | **1.00550004** |  |

Summary of model parameters for each of the categorical models:

| Model 1: FULL MODEL (categorical model) | Estimate | Incidence Rate Ratio (reference early lactation) | SE | *t* - value |
| --- | --- | --- | --- | --- |
| Intercept | 5.28232 |  | 0.13192 | 40.040 |
| Time of day (z-transformed) | -0.74215 |  | 0.07272 | -10.206 |
| Reproductive state (cycling) | -0.59220 | 1.69 | 0.14788 | -4.005 |
| Reproductive state (mid lactation) | -0.31697 | 3.13 | 0.11245 | -2.819 |
| Reproductive state (late lactation | -0.40855 | 2.44 | 0.13704 | -2.981 |

| Model 1: REDUCED MODEL, excluding time of sample collection (categorical model) | Estimate | SE | *t* - value |
| --- | --- | --- | --- |
| Intercept | 4.5642 | 0.1303 | 35.029 |
| Reproductive state (cycling) | -0.6429 | 0.1708 | -3.764 |
| Reproductive state (mid lactation) | -0.2966 | 0.1257 | -2.359 |
| Reproductive state (late lactation | -0.5048 | 0.1572 | -3.211 |

| Model 1: REDUCED MODEL, excluding reproductive state (categorical model) | Estimate | SE | *t* - value |
| --- | --- | --- | --- |
| Intercept | 4.93453 | 0.09898 | 49.86 |
| Time of day (z-transformed) | -0.74215 | 0.07389 | -10.10 |

Comparison of the two competing “continous” models: weighted AICc scores indicate that the full model fits the data best.

| **MODEL 2** | **AICc** | **delta AICc** | **expAICc** | **wAICc** |
| --- | --- | --- | --- | --- |
| full | 792.9065 | 0 | 1 | 0.90 |
| excluding reproductive state | 797.2306 | 4.3241 | 0.115088947 | 0.10 |
|  |  |  | **1.115088947** |  |

Summary of model parameters for each of the categorical models:

| Model 2: FULL MODEL (continous model) | Estimate | SE | *t* - value |
| --- | --- | --- | --- |
| Intercept | 4.92031 | 0.11704 | 42.041 |
| Time of day (z-transformed) | -0.67268 | 0.07872 | -8.545 |
| Age of offspring (z-transformed) | -0.39641 | 0.13044 | -3.039 |

| Model 2: excluding offspring age (continous model) | Estimate | SE | *t* - value |
| --- | --- | --- | --- |
| Intercept | 4.86725 | 0.10614 | 45.858 |
| Time of day (z-transformed) | -0.67944 | 0.07927 | -8.571 |
